# A vortex-based hydrodynamic cavitation manufacturing platform to generate albumin microbubbles for delivery of chemotherapies to cancerous tumours

**DOI:** 10.1016/j.ultsonch.2025.107350

**Published:** 2025-04-16

**Authors:** Promita Bhattacharjee, Abhijeet H. Thaker, Pratik Kumar Patel, Vivek V. Ranade, Sarah P. Hudson

**Affiliations:** a Department of Chemical Sciences, Bernal Institute, University of Limerick, Ireland; b Multiphase Reactors and Intensification Group Bernal Institute, University of Limerick, Limerick V94T9PX, Ireland; c SSPC, The Research Ireland Centre for Pharmaceuticals, University of Limerick, Ireland

**Keywords:** Microbubbles, Cavitation device, Curcumin, Drug delivery, Breast cancer

## Abstract

A novel approach was developed to create stable protein-based microbubbles using a vortex-driven hydrodynamic cavitation device. Such microbubbles, tiny gas-filled spheres, combined with ultrasound, can enhance drug uptake leading to inhibition of cancerous cell growth, boosting the effectiveness of anti-cancer drug molecules. The optimal conditions for the fabrication of stable bovine serum albumin (BSA) microbubbles were found to be a 15 wt% bovine serum albumin (BSA) solution at 60 °C with a pH of 6 and an ionic strength of 1.0 M. This resulted in stable BSA microbubbles with an approximate diameter of 7 μm. Curcumin-encapsulated BSA microbubbles (CBMs, 63 ± 1 μM curcumin per 10^1^⁰ microbubbles) were created using these optimised fabrication parameters as a model system for delivering chemotherapeutic agents. The maximum percentage of curcumin release from the CBMs into phosphate buffered saline with sonication (85 %) was significantly greater than without sonication (24 %). These microbubbles were then tested to assess their effectiveness in delivering curcumin to triple-negative breast cancer cells (MDAMB-231) using a cell-to-MB ratio of 1:100, an ultrasound intensity of 0.5 W/cm^2^, and an ultrasound exposure time of 10 s to maximise uptake. Kinetic studies demonstrated a significant enhancement in the uptake of curcumin by MDAMB-231 cells when encapsulated into the microbubbles with ultrasound application. A substantial reduction in cellular proliferation was observed in both 2D cell culture and 3D tumour spheroid models when MDAMB-231 cells were exposed to microbubbles loaded with curcumin and ultrasound was applied. The vortex-based hydrodynamic cavitation device successfully generated curcumin loaded microbubbles with a long shelf life (120 days at 4 °C), mild preparation conditions, and enhanced uptake into cancerous tumour spheroid models. This data demonstrates the potential of this device for the commercial manufacture of drug loaded microbubble-based delivery systems.

## Graphical abstract

**Figure:**
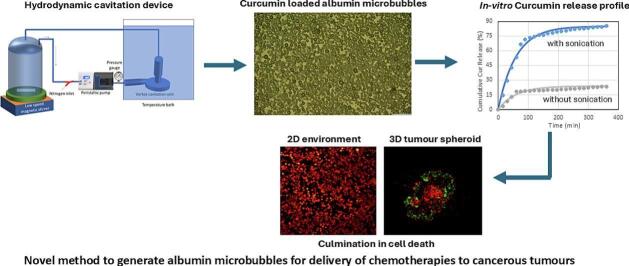

## 1 Introduction

The use of non-targeted drugs in cancer treatment often leads to significant off-target effects, which can harm healthy tissues [1]. To overcome this issue, ultrasound (US) techniques and microbubbles (MBs) have been developed as a promising solution. These methods enable the safe, efficient, and targeted delivery of anti-cancer drugs through US mediated drug delivery, reducing damage to healthy cells [1,2]. When exposed to US, microbubbles expand and contract with the US waves, generating an acoustic backscatter. This phenomenon not only aids in diagnostic imaging of organs [[3], [4], [5]] but also enhances their application in targeted drug delivery [[6], [7], [8], [9], [10]]. A commonly utilized form of gaseous microbubble in drug delivery comprises a gas core ranging from 2 to 10 µm, enveloped by a shell composed of stabilizing molecules. The stabilizing molecules may be surfactants, lipids, polymers, proteins or, some other, suitable, biocompatible materials [4]. Drug loading on MBs is achieved either by binding on the MB surface [11,12] or by encapsulation within the gaseous core as free drug [13] or within their shell (polymeric/liposomal) [14]. Alternatively, microbubbles can be co-administered with anticancer drugs to improve therapeutic effects [15]. For the latter, the MB surface can also be functionalized, using a targeting ligand. The drug can then be released when the MBs fracture and/or cavitation is induced by ultrasound at the desired target site. Even for delivering drug molecules through the blood–brain barrier, MBs can be a suitable facilitator [16].

Consequently, microbubbles composed of biocompatible materials find diverse applications in biomedical technologies. While polymers can yield stable MBs, their rigid nature may limit the impact of ultrasound on their use for effective drug delivery or for contrast in imaging [4]. Since the late 20th century, MBs made of lipids and proteins have been utilized in contrast imaging [17] as well as in drug delivery and for gene therapy [18]. Researchers have previously utilized serum albumins, either human or bovine, in the preparation of microbubbles [[20], [21], [22]]. Grinstaff and Suslick illustrated that heating serum albumin to its’ denaturation temperature, broke down the intramolecular disulfide bridges, releasing free –SH groups [21]. Following the heating process, sonication was used to emulsify the gas present in the solution. This step promoted the creation of new intermolecular disulfide bonds, which are essential for forming the shell of the MBs. Bovine serum albumin (BSA) has been widely used as a foundational protein in the fabrication of such MBs [8,22,23].

**Scheme 1:**
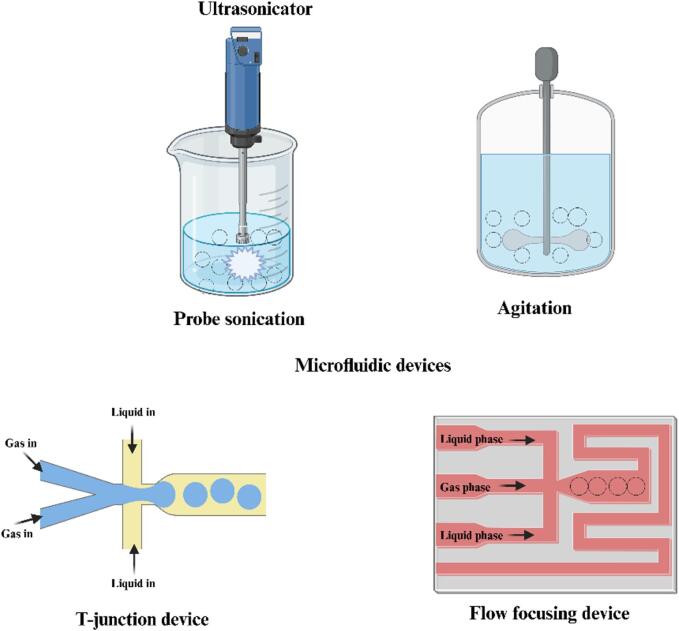
Commonly used methods of producing microbubbles. Created using Biorender.

Different technologies may be employed for producing MBs, e.g., probe sonication [8], agitation [9,10], and microfluidic devices [24], Scheme 1. However, the effectiveness of microbubbles diminishes with increasing polydispersity of the microbubble population [19]. Microfluidic devices (MFDs) can produce monodisperse MBs due to their unique mixing profiles [25,26]. Microfluidic techniques frequently generate microbubbles at relatively low yields, with large diameters (>100 μm), and require a complex setup [27]. Two primary types of microfluidic devices have been used in fabricating MBs: (i) T-junction devices, created by assembling together capillaries of predetermined diameters [28] and (ii) flow-focusing devices fabricated using soft lithography techniques [29,30].

More recently, the demand for hydrodynamic cavitation has grown in small-scale applications. This change was driven by a rise in the requirements for MFDs that can utilize small-scale cavitation [31]. Hydrodynamic cavitation (HC) generates vapour cavities, which grow, and collapse [32,33]. This repeated growth and collapse creates strong shear and high-velocity jets. The shear and high velocity provide the means for rupturing air bubbles and creating MBs. Recently, Thaker and Ranade experimentally and numerically demonstrated the potential of a vortex-based HC device (VHCD) to form microemulsions [34]. This device has the potential to produce monodisperse microbubbles. There are limited reports on the fabrication of gas microbubbles using cavitation based devices. One such continuous approach used a high gas concentration and resulted in a moderately polydisperse volumetric bubble size distribution within a tunable microbubble diameter range of 10–70 μm [35]. In another study, the dynamics of cavitation bubbles in a microfluidic channel under actuations were studied [36]. These two studies focused on the generation and behaviour of gas bubbles in water in cavitation devices but not on the fabrication of stable microbubbles composed of biocompatible materials. To the best of our knowledge, the work presented here is the first report of protein based monodisperse microbubbles fabricated using a cavitation based device.

Curcumin (Cur), a naturally occurring anti-cancer molecule derived from turmeric rhizomes, is an example of a model chemotherapy that exhibits poor aqueous solubility and low bioavailability [37]. Such molecules require an effective delivery system to ensure activity against cancer cells. Zhu et al. [38] established that curcumin-loaded poly(L-lactide-co-glycolide) microbubbles, fabricated using sonication, were toxic to liver cancer cells with both photodynamic and ultrasound therapies. The PLGA microbubbles demonstrated certain drawbacks, such as, large sizes, low capacity for drug loading, short times over which they may be circulated, non-targeted uptake by the reticuloendothelial system and challenges in crossing the blood–brain barrier [39,40]. An alternative approach to avoid low drug loading capacity involves using microbubbles co-administered with the chemotherapy. Wang et al tried this using curcumin with commercially available microbubbles to treat MDA-MB-231 cancer cells [15]. In the work presented here, microbubbles, blank and loaded with curcumin, were fabricated using a VHCD, with BSA as the protein shell and air as the core gas. Process parameters like BSA concentration, preheat temperature and pH and ionic strength of the precursor BSA solution were optimised to produce MBs with a narrow size range (monodisperse). Microbubble stability during storage and freeze drying was also studied. The ultrasound-assisted cumulative release of curcumin and its uptake by triple-negative breast cancer cells (MDAMB-231), when loaded onto MBs and co-administered were comprehensively studied. In addition, the effectiveness of the curcumin loaded microbubbles on the inhibition of cellular proliferation in 2D monolayer and 3D spheroid models with MDAMB-231 cells was explored.

## 2 Materials and methods

### 2.1 Materials

Bovine serum albumin (BSA; Purity: >= 98 %), foetal bovine serum (FBS), hydrochloric acid (HCl; 37 %), sodium hydroxide (NaOH; Purity: >= 98 %), sodium chloride (NaCl; Purity: >= 99.5 %), N-acetyl-DL-tryptophan (Tryp; Purity: >= 99 %), trypsin-EDTA, curcumin (Purity: = 98 %), penicillin–streptomycin (Pen/Strep), and Dulbecco's Modified Eagle Medium/Nutrient Mixture F-12 (DMEM/F-12) were used as received from Sigma Aldrich. Trypsin phosphate versene glucose (TPVG) purchased from Lonza. Live/Dead assay kit (Invitrogen) procured from Thermo Fisher Scientific, Ireland. Triple-negative breast cancer cells, MDAMB-231, procured from American Type Culture Collection. Where it has not been explicitly specified to be otherwise, the chemicals and reagents were procured and used as received, from Sigma-Aldrich.

### 2.2 Methods

#### 2.2.1 Process parameter optimisation for microbubble formation

The fabrication of microbubbles (MBs) involved the preparation of precursor BSA solutions in accordance with a previously established protocol, with modifications [22]. Bovine serum albumin (BSA) and Tryp (9.8 mg/mL) were dissolved, using demineralized water, at varying BSA concentrations (w/w): 10 %, 12 %, and 15 %. The solution thus created was stirred for a minimum of 2–3 h at 60 °C until Tryp completely dissolved, resulting in a transparent solution. Subsequently, to further optimise the solution pH, its ionic strength and the preheat temperature, pH was adjusted to between 3.0 and 8.0, by adding HCl (pH 7.0 to 3.0) or NaOH (for pH 8). The ionic strength was standardized using NaCl, at 0.1 M, 0.5 M, and 1.0 M. The solutions were preheated at five different temperatures, 25 °C, 35 °C, 45 °C, 55 °C, and 60 °C, for 10 min each, in a water bath.

**Fig. 1:**
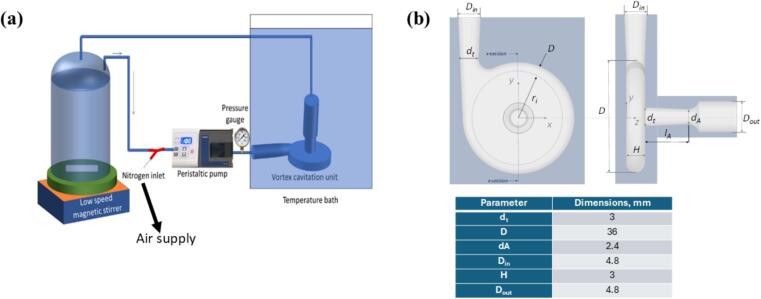
(a) Schematic of the vortex-based hydrodynamic cavitation device (VHCD) used in the current work and (b) its geometric details.

Microbubbles were generated using a vortex based hydrodynamic cavitation device (VHCD). The unit is based on a design of Vivira Process Technologies and improved as per the patent of Ranade et al [41]. The VHCD throat diameter (dT) was 3 mm. Other dimensions of the VHCD were determined with reference to dT and the proportions were the same as reported by Simpson and Ranade [42]. The VHCD set-up and geometric details are shown in Fig. 1. The total working volume of the loop was ∼57 ml. The VHCD volume was 0.8 ml. The volume of tubing used was 56 mL (Longer LP-PLATINUM-35# silicon tube; length: 115 cm; ID: 7.9 mm). The solutions underwent up to 200 passes through the VHCD. Pressure drop (ΔP) across the solutions was 250 kPa. To generate the microbubbles, air (at 1 mL/min) was injected into the system. The solutions thus created had a total volume of 60 mL. Three BSA concentration, 10, 12 and 15 wt%, were used to produce the microbubbles. Once prepared, the microbubble dispersions were placed in sealed glass vials and stored either at 4 °C or at room temperature (20–25 °C).

#### 2.2.2 Microbubble size and number

Microbubble counting and sizing was conducted as per the protocol in a previously published work [43]. The air-core diameter of the microbubbles was determined through image analysis, using Fiji Image J2, Windows 64-bit (National Institutes of Health, Bethesda, MD, USA), n = 50. A 10 μL sample of the microbubble suspension was placed onto a Neubauer improved cell counting chamber (Hausser Scientific Company) and covered with a 24 mm × 24 mm glass coverslip. Using a bright-field microscope (Olympus) fitted with a camera, at least 10 images of the microbubbles were captured at 40 × magnification.

The number of microbubbles (*N_MB_*) at each BSA solution concentration (*w*) was fitted to the following equation:

(1) $$N_{MB}=N_{\max}\left[1-e^{-0.5\left(w-w_{c}\right)}\right]$$

And values for the two fitting parameters, the maximum number of bubbles, *N_max_*, and the critical BSA concentration, *w_c_*, were determined for the microbubble dispersion immediately after preparation and after 30 days storage at 4 °C.

The reduction in bubble diameter (*d_B_*) over time was expressed as a first order decay:

(2) $$d_{B}=d_{B0}\;e^{-kt}$$

In Equation 2, k is the first order rate constant for the decrease in MB diameter, over time.

The reduction in number of microbubbles (*N_B_*) over time was also expressed as a first order decay:

(3) $$N_{B}=N_{B0}\;e^{-kt}$$

In Equation 3, k is the first order rate constant for the decrease in MB number in the suspension over time and under different pH and ionic strength conditions.

#### 2.2.3 Shell thickness of fabricated MBs

Fluorescein isothiocyanate (FITC) at a concentration of 10 µM, was introduced into a known quantity of microbubbles (10^5^/mL). This mixture was then incubated at 4 °C, in the dark, for 12 h. The suspended microbubbles were rinsed thrice to wash out unbound dye, using MilliQ water to re-suspend the MBs. After washing, the microbubble suspension was kept in an Eppendorf, in a vertical position, in the dark. After leaving the suspension undisturbed for two hours at 4 °C, the microbubbles had risen to the top, due to density differences, and formed a thin, surface layer. From this thin layer ∼50 µl was placed on a microscopic slide to examine. The shell thickness of fluorescently labelled microbubbles was assessed using the Image Xpress® Micro Confocal Microscope (Molecular Devices), followed by image post-processing with Fiji (Image J) software. A sensitivity analysis was conducted for the shell thickness variation with the number of MBs (n = 50).

#### 2.2.4 Freeze-drying of microbubbles

The prepared MB dispersions were stored at 4 °C for 30 days. Subsequently, MB dispersions were dialyzed, eliminating any free protein in the solution. The dialysis membrane used had a pore size of 100 kDa (Spectrum Laboratories, Inc., Rancho Dominguez, CA, USA). The membrane tubes containing the microbubble dispersion were immersed in demineralized water within a 5 L bucket. The bucket was then placed in a room at 4 °C. The bucket contents were slowly stirred using a magnetic stirrer. The water was replaced following three hours of stirring. The process was repeated five times. Following dialysis, the MBs were freeze-dried (Freeze-drier of Telstar, LyoQuest) for a 2-day period, at −85 °C and 0.080 mbar.

Microbubble morphology was characterised through scanning electron microscopy (SEM − Hitachi SU-70) imaging. The freeze-dried MBs were affixed onto a silver tape. This tape was positioned onto an aluminium holder, using a piece of conductive, carbon tape. To facilitate drying of the sample, it was then subjected to vacuum. For MB dispersions, adherence to poly-lysine-coated glass surfaces occurred, followed by immersion in glutaraldehyde for enhanced fixation. Subsequently, the MB samples were treated with acetone, followed by air-drying. All the samples underwent gold sputtering to generate an approximately 10 nm gold coating.

#### 2.2.5 Production and morphology of Cur-loaded microbubbles (CBMs)

BSA (15 wt%) and Tryp (0.1 wt%) were dissolved in deionized water, method as described in Section 2.2.1. Cur-loaded microbubbles (CBMs) were fabricated following the established protocol with modifications [44]. Briefly, an excess of curcumin (approximately 12.67 wt%) was added to ethanol, at room temperature. This mixture was stirred continuously for about 1 h, when it was taken and centrifuged at 14000 rpm, over 30 min. Using a syringe filter (filtration specification 0.2 µm), the supernatant, consisting of a saturated curcumin solution, (3 mL) was slowly added to 57 mL of the protein solution. A 5:95 vol ratio was maintained to prevent curcumin precipitation. The resulting BSA-Cur solution was then stirred for approximately 4 h, at a rate of 500 rpm, resulting in a clear, transparent solution with no observed precipitation of curcumin or BSA. Adjustments of the protein solution to pH 6 and ionic strength (1.0 M) were made as described in Section 2.2.1.

With continuous stirring at 500 rpm, the solution was heated to reach 60 °C. This solution was then passed (200 passes) through the cavitation device, Fig. 1, to generate monodisperse microbubbles.

For the subsequent experiments and characterisation, after fabrication, the curcumin loaded BSA microbubble (CBM) dispersion (5 mL) was taken in a Falcon tube and subjected to 10 min of centrifugation (14000 rpm). Following centrifugation, the microbubbles had collated at the surface and from here, they were carefully collected and washed with DI water three times (5 min each time) to reach a 10^10^ microbubbles/mL MBs concentration. These MBs were stored at 4 °C. The morphology of curcumin-loaded microbubbles was examined using a bright field microscope (Olympus) equipped with a camera.

#### 2.2.6 Quantification of Cur-loaded on the microbubble surface

After fabrication and washing as described in section 2.2.5, the curcumin –BSA microbubbles (∼10^10^ MBs; approx. 120 µl solution) were accurately introduced into 1 mL of ethanol. They then went through another round of centrifugation (14000 rpm) for 10 min. Addition of ethanol dissolved any curcumin loaded on the surface of the MBs. Concentration of curcumin dissolved in ethanol was assessed based on UV absorbance (at 425 nm) characteristics of the supernatant obtained following centrifugation, using a calibration curve (Fig. S1) [44]. The presented results are the average of 3 experiments.

#### 2.2.7 *In vitro* release of curcumin from microbubbles

The kinetics of Cur release from the surface of the MBs, with and without sonication, was investigated using UV–vis spectrophotometry, four weeks after their preparation. From the suspension of curcumin loaded MBs prepared in section 2.2.5, a calculated volume of the microbubble dispersion was taken to prepare two samples, each containing 6 × 10^7^ of the Cur loaded BSA MBs, in 5 mL PBS solution (kept at 37 °C). One of these samples underwent sonication for 10 s with an ultrasonic bath sonicator (Branson 3800, frequency: 40 kHz, power dissipation: 130 W), intensity 0.5 W/cm^2^, one exposure. The other sample did not undergo sonication. A volume of ∼30 μL was extracted from each sample at 15-minute intervals and analysed by UV–vis spectroscopy. Concurrently, 30 μL PBS was put into each solution so that they were maintained at a constant volume. This sampling and subsequent analysis was conducted over a period of 6 h. The Cur cumulative release was assessed using UV absorbance measurements at 425 nm wavelength.

(4) $$\mathrm{Cumulative}\;\;release_{n}\left(\%\right)=\;\frac{\sum_{i=1}^{n}C_{i}}{C_{0}}\times 100$$

Where C_i_ is the concentration measured at each time point and C_0_ is the known initial concentration of curcumin calculated from section 2.2.6.

The cumulative release of curcumin was then fitted to first order release kinetics:

$Cumulativerelease\left(\%\right)=P_{\max}\left(1-e^{-k_{r}t}\right)$(5)

In Equation 5, $k_{r}$ is the first order rate constant for the release and $P_{\max}$ is the maximum percentage release.

#### 2.2.8 Chemotherapeutic activity of Cur-loaded microbubbles (CMBs)

##### 2.2.8.1 Optimization of cell to microbubble ratio

Triple-negative breast cancer cells, MDAMB-231, were cultured using DMEM/F-12 culture media. This media was supplemented with 10 % FBS and 1 % pen/strep. The culture was conducted in a controlled, humidified environment (5 % CO_2_, 37 °C) until cells reached 90 % confluency. Following trypsinization, MDAMB-231 cells were taken and seeded in six-well plates. Following seeding, the cells were incubated for 24 h.

To optimize the cell to microbubble ratio, three cell-to-CBM ratios were studied: [100:1 (cell:MB = 10^12^:10^10^), 1:1 (cell:MB = 10^10^:10^10^), and 1:100 (cell:MB = 10^8^:10^10^)]. All samples underwent sonication with 1 MHz frequency. Sonication intensity was 0.75 W/cm^2^ but seven exposure times were trialled: 5, 10, 20, 30, 40, 50, and 60 s. Cells which had not been introduced to MBs or sonication served as controls. Following sonication, samples were taken and incubated for another 24 h.

After this incubation period of 24 h had been completed, all the cells were rinsed with PBS followed by 2 min of treatment with 1 mL of TPVG. The TPVG was subsequently decanted off and to each well, a millilitre of PBS was added. Scrappers were used to collect the cells in 2 mL centrifuge vials. Cells in the vials were then centrifuged for 10 min at 1500 g. Discarding the supernatant, the resulting cell pellet was re-suspended into 500 μL of methanol. The pellets then went under a probe sonication cycle –10 s ON, 20 s OFF, repeated three times – to lyse the cells and extract curcumin into the methanol solution. For the lysed cell mixture, there was a further round of centrifugation of 10 min (1500 g). Following this centrifugation, the supernatant formed was placed into a flat-bottom, 96-well, black polystyrene plate. Fluorescence measurements were done on this supernatant. Curcumin uptake was measured based on the fluorescence intensity of the methanol solution containing the extract: excitation wavelength of 423 nm and emission wavelength of 570 nm.

##### 2.2.8.2 Optimization of ultrasound intensity and exposure time

MDAMB-231 cells were cultured following the protocol described above. Following trypsinization, MDAMB-231 cells were seeded into six-well plates. They were incubated for 24 h in a humidified atmosphere, maintained at 37 °C and CO_2_ levels held consistently at 5 %. For all samples, cell-to-CMB ratio was maintained at 1:100 (cell:MB = 10^8^:10^10^) and ultrasound exposure times in this step were kept to 10, 20, and 30s.

All experimental groups underwent sonication with a 1 MHz frequency at ultrasonic intensities of 0, 0.25, 0.5, and 0.75 W/cm^2^. Cells without sonication served as controls. Following the sonication step, samples underwent another 24 h of incubation. Subsequently, using a fluorescence reader, fluorescence intensity was measured, at the emission wavelength of 570 nm, as described in the protocol above. Cells, not treated with curcumin or microbubbles and with or without ultrasound, served as controls.

##### 2.2.8.3 Delivery efficiency and cytotoxicity of Cur loaded BSA MBs (CMBs) to triple-negative breast cancer cells

Table 1 presents a list of samples tested for curcumin uptake and cytotoxicity. For each of the experimental groups, optimized operational delivery parameters were applied. These were: cell:MB ratio of 10^8^:10^10^, an ultrasonic intensity of 0.5 W/cm^2^, and exposure time to ultrasound of 10 s. Subsequently, both treated and untreated cells underwent a 24-hour incubation. Following the incubation period, fluorescence intensity from the lysed cells was measured following the protocol described above. The microbubble quantity in each experimental group was maintained at a constant 10^10^/well for the delivery efficiency studies. Additionally, the curcumin concentration in each sample was standardized to 63 μM, equivalent to the amount of curcumin loaded on 10^10^ CMBs.

**Table 1:** List of experimental groups tested to determine the impact of microbubbles (formed from a 15 wt% BSA solution) on viability and curcumin uptake by triple-negative breast cancer cells, MDAMB-231. *63 µM curcumin in dimethyl sulfoxide was added to each well.

**No.**	**Experimental group**	**Details**
1.	Control	Untreated (no curcumin or microbubbles)
2.	Free Cur	Treated free, dissolved curcumin* but no microbubbles
3.	Free Cur MBs	Treated with free dissolved curcumin* and BSA microbubbles
4.	Cur-loaded BSA MBs (CMBs)	Treated with Cur-loaded BSA MBs

##### 2.2.8.4 Cytotoxicity

The assessment of cytotoxicity under different conditions was conducted using a 2D culture, with the MDA-MB-231 breast cancer cells. Cell seeding took place using 10^6^ cells/mL into a 6-well plate. The seeded cells, in culture media, were maintained overnight in an incubator at 37 °C, 90 % RH, and 5 % CO_2_. After a 24-hour incubation period, cell confluency had reached ∼90 %. At this point, curcumin loaded microbubbles, CMBs were introduced into the cell culture plate wells. The CMBs were added at a consistent concentration of ∼10^8^ microbubbles/well, to each well. There were four experimental groups at this point: 1 (with ultrasound), 2 (with/without ultrasound), 4 (with/without ultrasound), and Control (Table 1). Addition of CMBs was done in a manner to maintain the cell-to-microbubble ratio at 1:100, ensuring Cur concentration was the same for all the samples. One plate underwent sonication for 10 s (intensity = 0.5 W/cm^2^). The other plate was not subjected to sonication. Subsequently, the cell culture plates were incubated for a 48-hour period, in a 37 °C, humidified atmosphere, with CO_2_ levels held at 5 %. Following incubation, both plates (sonicated and not-sonicated), underwent the Alamar blue assay (Invitrogen), following the guidelines provided by the manufacturer. Cell viability was also assessed using the fluorescence, live/dead assay (Invitrogen). This process also followed the protocol provided by the manufacturer. After a 48-hour incubation cell media as well as the microbubbles were taken out of the wells. The wells were then incubated using a 2 μM calcein AM and 4 μM ethidium homodimer-1 mix, for a period of one hour, at 37 °C in the dark. The cells interacted with the fluorescence dyes. Representative images, with green (live) and red (dead) cells, were captured using the Image Xpress® Micro Confocal Microscope (Molecular Devices). Subsequently, image post-processing was performed using Fiji (Image J) software.

#### 2.2.9 Assessment of cell viability in a 3D spheroid model

Three dimensional spheroids, using MDA-MB-231 cells, were created through the hanging-drop culture method [45], utilizing rat tail collagen I (Corning) as the extracellular matrix (ECM). A T25 flask with cells at 85 % confluency underwent trypsinization (1 mL of trypsin). Subsequently, 4 mL of complete DMEM/F-12 medium, freshly prepared, was added and the solution was mixed well. After centrifugation for 10 min, at 734 g, the obtained pellet was extracted. This pellet was then resuspended in 4 mL of fresh complete medium. Cells, ∼ 35 μL, from the medium (∼6 × 10^3^ cells) were added onto the inner surface of the petri dish lid, in the form of drops. To create a moist environment for spheroid growth, 30 mL, 1X PBS was provided at the base of the Petri dish. The lid of the Petri dish, which had the droplets containing cells on it, was then taken and put upside down, covering the base. Spheroids were grown until they were well-formed. At the 36th hour, the spheroids were harvested.

Each hanging drop represented one tumour spheroid. The harvested spheroids were dispersed in to 50 μL of ECM-complete medium (2:1 vol ratio), using a 12-well plate matrix. These spheroids were subsequently treated with CMBs and curcumin dissolved in DMSO with and without sonication, maintaining a constant cell/microbubble ratio, i.e., 1:100. Cells treated with ultrasound, but without any exposure to curcumin or MBs, were the control. Sonication time (10 s) and intensity (0.5 W/cm^2^) were also held constant.

The culture plates then underwent a 24 h incubation, in the environment maintained at 37 °C and 5 % CO_2_ level, in a humidified atmosphere. Following incubation, the plates (treated with/without sonication) underwent live/dead assay (Invitrogen), as per the instructions from the manufacturer (details in section 2.2.8). Live cells were stained with calcein AM (Coloured green, wavelength of 488  nm). Dead cells were stained with ethidium homodimer-I (Coloured red, wavelength of 543 nm). The stained constructs were examined with an Image Xpress® Micro Confocal Microscope (Molecular Devices). The images obtained were processed using Fiji (Image J) software.

#### 2.2.10 Statistical analysis

For the comparison of results obtained with various experimental group, a one-way ANOVA was employed. Where the ANOVA tests pointed to a significant difference, Tukey's post-hoc HSD test was used for pairwise comparisons, using the statistical platform R. Significant differences were denoted as follows: ***p < 0.001; **p < 0.01; *p < 0.05. All data, unless otherwise specified, has been presented as mean ± standard deviation (SD), with sample size = 3.

## 3 Results and discussion

### 3.1 Microbubble (MB) fabrication

Protein solutions were initially prepared at a range of BSA concentrations (10 – 15 wt%) in deionised water. Subsequently, process parameters were screened to determine the optimal conditions that would produce stable, monodisperse BSA microbubbles using the vortex based hydrodynamic cavitation device (VHCD), Fig. 1. Hydrodynamic cavitation involves the formation and collapse of cavities in a liquid flow. The collapsing cavities generate intense shear forces, very high localised energy dissipation rates and shock waves [32]. These intense shear, dissipation rates and shock waves cause breakage of injected gas bubbles and generate micro bubbles.The VHCD used in this work has previously been used for generating small droplets [46,47] and gas bubbles [48]. Thus, the optimized VHCD design and operating parameters from these previous studies were used and the following parameters for the production of BSA microbubbles were screened: pre-heat temperature (20 to 60 °C), pH value (4 to 8) and ionic strength (0.1 to 1.0 M).

### 3.2 Morphology and size distribution of fabricated MBs

**Fig. 2:**
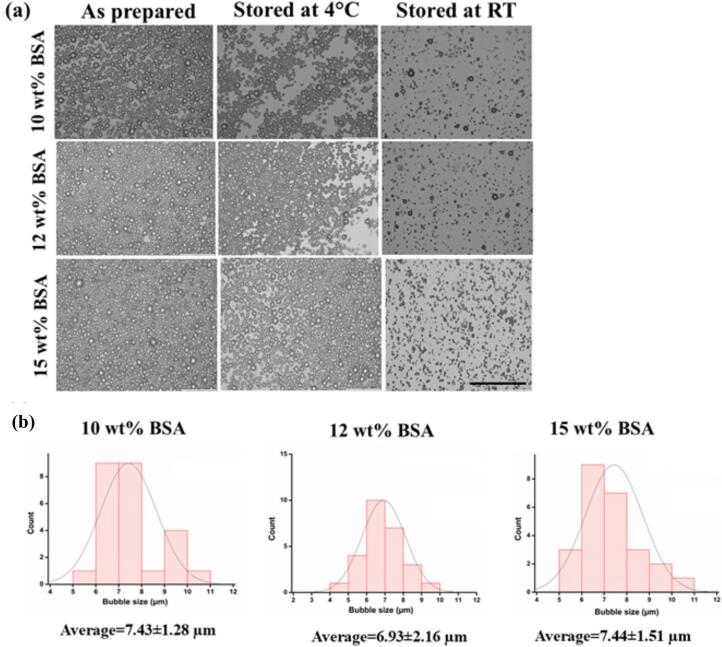
(a) Optical micrographs of microbubbles (MBs) fabricated from different aqueous BSA solutions (10 wt%, 12 wt% and 15 wt%); immediately after preparation, MBs stored at 4 °C, MBs stored at room temperature for 30 days. Scale bar, Fig. 2 a, 100 µm. (b) MBs size distribution (air-core diameter), immediately post preparation.

When BSA was dissolved in water and heated to 60 °C before flowing into the HC unit, varying the BSA concentration from 10–15 wt% had no significant impact on the air-core diameter of the microbubbles (∼6.93–7.44 µm), Fig. 2. From the images in Fig. 2(a), it is evident that over time, the number of microbubbles that dissipated was lower when stored at 4 °C, as opposed to at room temperature. The temperature-dependent nature of microbubble stability can be attributed to the reduction in shell rigidity with increasing temperature, likely due to relaxation or creep. [22].

### 3.3 Effect of BSA concentration and temperature on stability, number and size of MBs

**Fig. 3:**
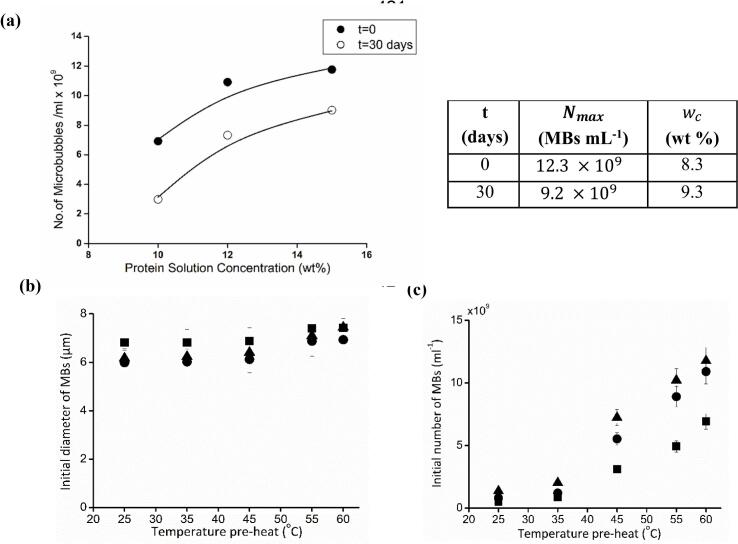
(a) Number of microbubbles /mL after preparation, preheat temperature = 60 °C and after storage at 4 °C for 30 days. Continuous lines indicate fitting to Equation (3) with parameter values after preparation and after 30-day storage (b) Preheat temperature’s impact on microbubble air-core diameter (c) number concentration, fabricated from 10 wt%,■; 12 wt%,●; and 15 wt%,▲ BSA solutions.

A significantly higher number (p < 0.05) of microbubbles were generated from 12 wt% and 15 wt% BSA solutions when compared with a 10 wt% BSA solution, Fig. 3(a). After storing the microbubbles at 4 °C for 30 days, MB numbers reduced at all BSA concentrations. A higher reduction (p < 0.05) in MB numbers was observed for the 10 wt% BSA solution when compared with 12 wt% and 15 wt% BSA solutions. This result indicates that microbubbles fabricated from 12 wt% and 15 wt% BSA solution are more stable than the microbubbles generated from 10 wt% BSA solution. The number of microbubbles present was expressed as $N_{\max}\left[1-e^{-0.5\left(w-w_{c}\right)}\right]$ (Equation 1) where reduction in maximum number of MBs ($N_{\max}$) and the increase of critical BSA concentration parameter ($w_{c}$) after 30 days was observed, Fig. 3(a). This implies that higher BSA concentrations (w) are required to sustain a defined number of microbubbles in the dispersion if they are not used immediately after fabrication and instead are left in storage at 4 °C. The microbubble shell can be conceptualized as a complex network of BSA molecules, forming multiple layers through thermally induced protein–protein interactions [22]. Regardless of the BSA solution concentration, the preheat temperature did not significantly affect the MB diameter, Fig. 3(b), but increasing the temperature of the solution from 25 to 60 °C led to an increase in the initial number of microbubbles at all protein concentrations screened, Fig. 3(c). Further increases beyond 60 °C did not increase the number of microbubbles further (data not shown).

### 3.4 Shell thickness, morphology and stability of MBs

**Fig. 4:**
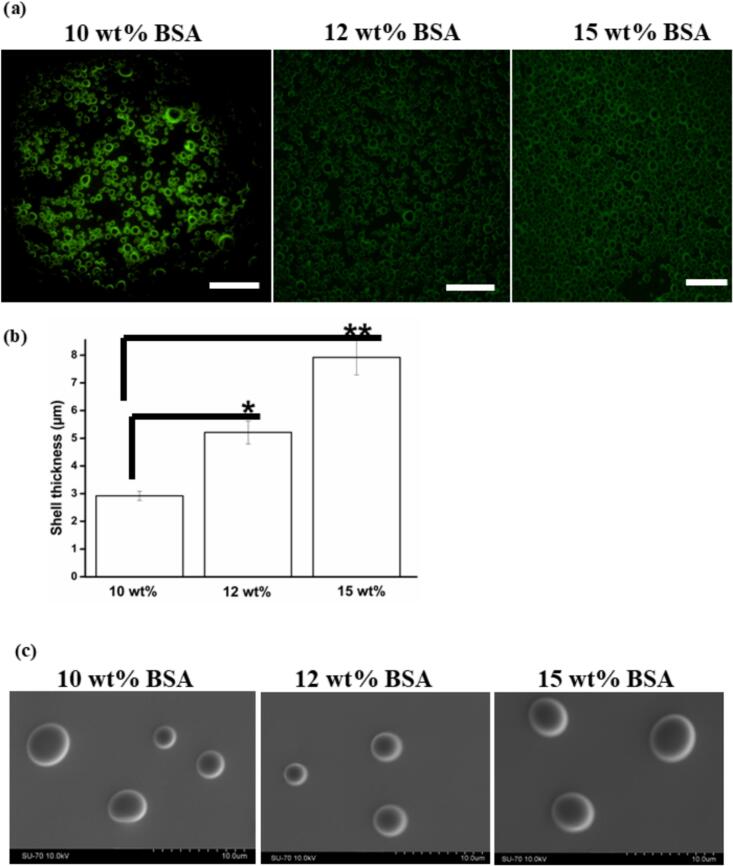
(a) FITC tagged microbubbles fabricated from BSA aqueous solutions (preheated at 60 °C) at 10 wt%, 12 wt% and 15 wt% concentrations. Scale bar = 50 µm, (b) shell thickness of microbubbles (n = 50) and (c) morphology of freeze-dried microbubbles. **p < 0.01; *p < 0.05.

Upon exposure to the fluorescent laser, the MBs prepared from 10 wt% BSA solution were unstable and burst rapidly while the MBs fabricated from 12 wt% and 15 wt% BSA solution were found to be more stable, Fig. 4(a). This finding confirms the higher stability of MBs fabricated from 12 wt% and 15 wt% BSA solution when compared with MBs fabricated from 10 wt% BSA solution. This result supports the finding of Rovers et al. [22] that higher protein concentrations lead to more stable microbubbles. Shell thickness of MBs varied linearly as the BSA concentration increased, Fig. 4(b). In this case, the MB shell may be conceptualized in the form of a complex net of BSA molecules, forming multiple layers through thermally induced protein–protein interactions [22]. Thus, the thickness of the shell emerges as a critical determinant for MB stability. At the submicron level, the cross-linked protein shell acts a barrier, holding the air within the bubbles. Hence, the bubble is not in a diffusive equilibrium with its surrounding medium. The process of microbubble coagulation is slow due to stabilization facilitated by protein chains extending from the shell into the bulk [49]. The investigation revealed that both shell thickness and stability, especially under laser exposure, improved with increasing BSA concentration. After freeze-drying, no noticeable change in microbubble morphology was observed. The redispersed MBs had the same morphology and size, Fig. 4(c) and Fig. S2, demonstrating the robustness of the MB formulation to drying, allowing for a long shelf life and ease of transport and storage.

**Fig. 5:**
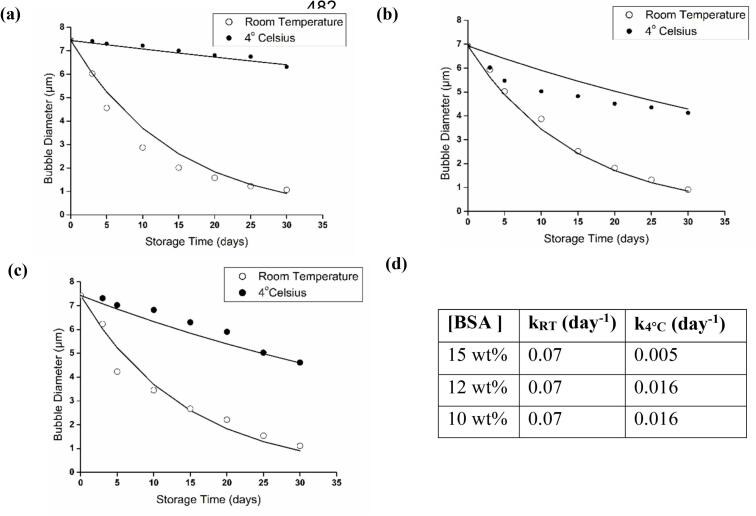
Average air-core diameter over time at 4 °C (filled circles) and room temperature (empty circles) for microbubbles fabricated from BSA aqueous solutions at 60 °C and concentrations of (a) 15 wt%, (b) 12 wt%, and (c) 10 wt% and (d) the decay rate constants from fitting to the first order rate equation (Equation 4).

As mentioned above, during characterisation, microbubbles prepared from 10 wt% BSA solution were observed to be unstable and burst rapidly while the microbubbles fabricated from 12 wt% and 15 wt% BSA aqueous solutions were found to be more stable, indicating the higher stability of microbubbles at the higher concentration, Fig. 5(a-c). A high protein content is favored when protein molecules adopt a compact conformation with minimal repulsion between them that leads to formation of more stable microbubbles [22]. Microbubbles stored at 4 °C exhibit greater stability than those kept at room temperature, as higher temperatures reduce the rigidity of the microbubble shell. The reduction in microbubble diameter was found to follow first order kinetics at all BSA concentrations tested, Equation 2, Fig. 5(d). With increasing protein concentration, the reduction in microbubble diameter was slower when stored at 4 °C (first order rate constant = 0.005 day^−1^ at 15 wt% and 0.016 day^−1^ at 10 and 12 wt%) than at room temperature (first order rate constant = 0.07 day^−1^ at all concentrations) over 30 days, Fig. 5 (d). Thus the 15 wt% BSA solution showing the highest stability at 4 °C.

### 3.5 Effect of pH and ionic strength on the stability of MBs

**Fig. 6:**
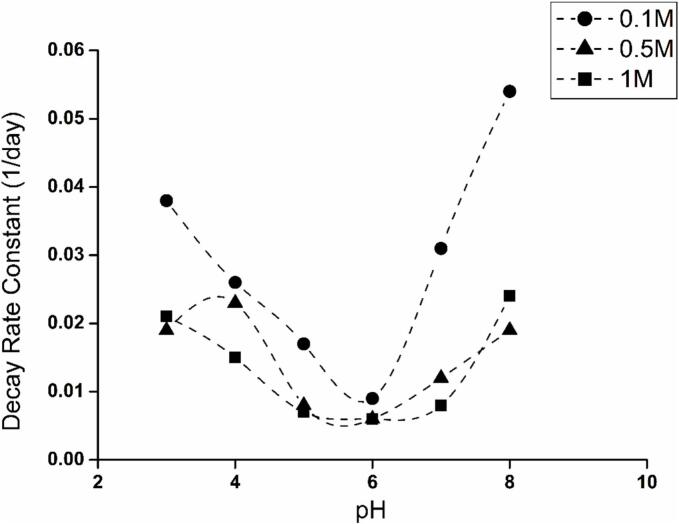
First order decay rate constants for the microbubble number concentration over time for 15 wt% BSA protein solutions at multiple ionic strength and pH points (pH 3–8): 0.1 M, 0.5 M and 1 M.

All of the above characterisation studies were conducted in deionised water but in order to use a microbubble dispersion *in vivo*, it would need to be suspended in an aqueous medium at a biocompatible pH and ionic strength to prevent irritation or pain to the patient. Thus, the stability and number of microbubbles generated from BSA solutions at a range of pH values and ionic strengths were explored. In this case, the first order decay rate constant (Equation 3) for the number of microbubbles in the dispersion is influenced by pH and shows a minimum at pH = 6 for all BSA concentrations and at all ionic strengths, Fig. 6 and Fig. S3. This pH lies above but near BSA’s isoelectric point (∼4.8), where the overall charge on the protein will be low. Thus the pH used during the fabrication of the microbubbles needed to be set close to the isolectric point. Moving too far above or below this pH value would result in a higher overall charge on the BSA molecules, resulting in a less compact MB shell, which was shown to destabilise the microbubbles, Fig. 6. The high yield and stability for the MBs near the isoelectric point, may be related to the extensive surface coverage with BSA and Tryp that can be achieved at the air–water interface when the involved protein molecules are carrying little charge [50]. Interestingly, adding NaCl, generating solutions with higher ionic strength, in our experiments, resulted in higher MB yields (Fig. S3). Greater rigidity at the air–water interface and higher surface coverage near the isoelectric point or with added salt has previously been ascribed to the charge on the BSA molecules and their conformation [51], [52].

*In vitro* imaging demonstrated that contrast enhancement was observed when the BSA microbubbles, even up to 120 days old, were injected into a dialysis bag (Fig. S4). The size distribution of CMBs did not change significantly after 120 days storage at 4 °C, Fig. S5.

### 3.6 Controlled release of curcumin from BSA microbubbles

The 15 wt% BSA solution, pre heated at 60 °C, at pH 6, and with an ionic strength of 1.0 M, was selected for the development of microbubbles loaded with a model anti-cancer drug (curcumin) due to its ability to generate microbubble dispersions in the VHCD with good stability. As discussed earlier, BSA microbubbles can be activated by ultrasound, resulting in controlled delivery drugs by applying pressure waves that induce echogenic cavitation of MBs and can target tissues in the body [53]. A significant economic advantage of such a drug delivery system lies in their utilization of ultrasonography. Ultrasound equipment is readily available in most clinics and hospitals (frequency range:1 MHz to 20 MHz, power: 200–400 Watts, intensity:1–10 mWatt/cm^2^) [54], [55]. The ultrasound-sensitive bubbles and drugs can be integrated into a single complex, heightening the effectiveness of US mediated drug delivery systems [56]. Thus, in this work, curcumin was selected as a readily available model chemotherapeutic with low solubility and permeability [57] to develop into a complex with BSA microbubbles.

**Fig. 7:**
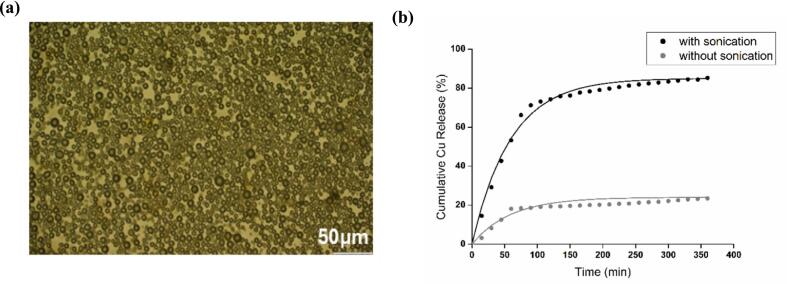
(a) Optical micrograph of CMBs and (b) % Cumulative curcumin release (calculated from Equation 1 and 2) from CMBs, in PBS, kept at 37 °C with (black) and without (grey) sonication.

Optical microscopy image of CMBs (Fig. 7(a)) revealed that their air-core diameter was in same range as the blank BSA microbubbles, ∼7.1 ± 1.44 µm (Fig. S5). The curcumin loading was determined as ∼63 ± 1 μM/10^10^ microbubbles (n = 3). With and without ultrasound, the value of the first order rate, $k_{r}$ for the release of curcumin from the microbubbles, was found to be the same, at 0.017 min^−1^, Equation 4. However, the maximum percentage release, $P_{\max}$ with sonication (85 %) is significantly higher than that without sonication (24 %). When a microbubble destabilises and the trapped gas in the bubble escapes, that is, when the microbubble bursts, the BSA shell also collapses and starts to dissolve. The curcumin molecules trapped within the BSA protein shell desorb from the protein molecules and diffuse out into the bulk solution. Without sonication, and over time, the CMBs are destabilizing and bursting, as shown in Fig. 5, Fig. 6, leading to a fraction of the total amount of curcumin loaded being released. The extent of this release, $P_{\max}$, depends on the number of microbubbles that have burst. With sonication, more microbubbles burst, increasing the $P_{\max}$ but the first order rate, $k_{r}$, dependent on the desorption and diffusion of curcumin away from the BSA protein molecules, is the same whether ultrasound is applied or not. Thus, the manufactured microbubbles effectively released curcumin from their surface upon exposure to ultrasound (Fig. 7(b)). The size distribution of CMBs was checked before use and had not changed significantly after 4 weeks storage at 4 °C, Fig. S5.

### 3.7 Curcumin uptake by triple-negative breast cancer cells, MDAMB-231

**Fig. 8:**
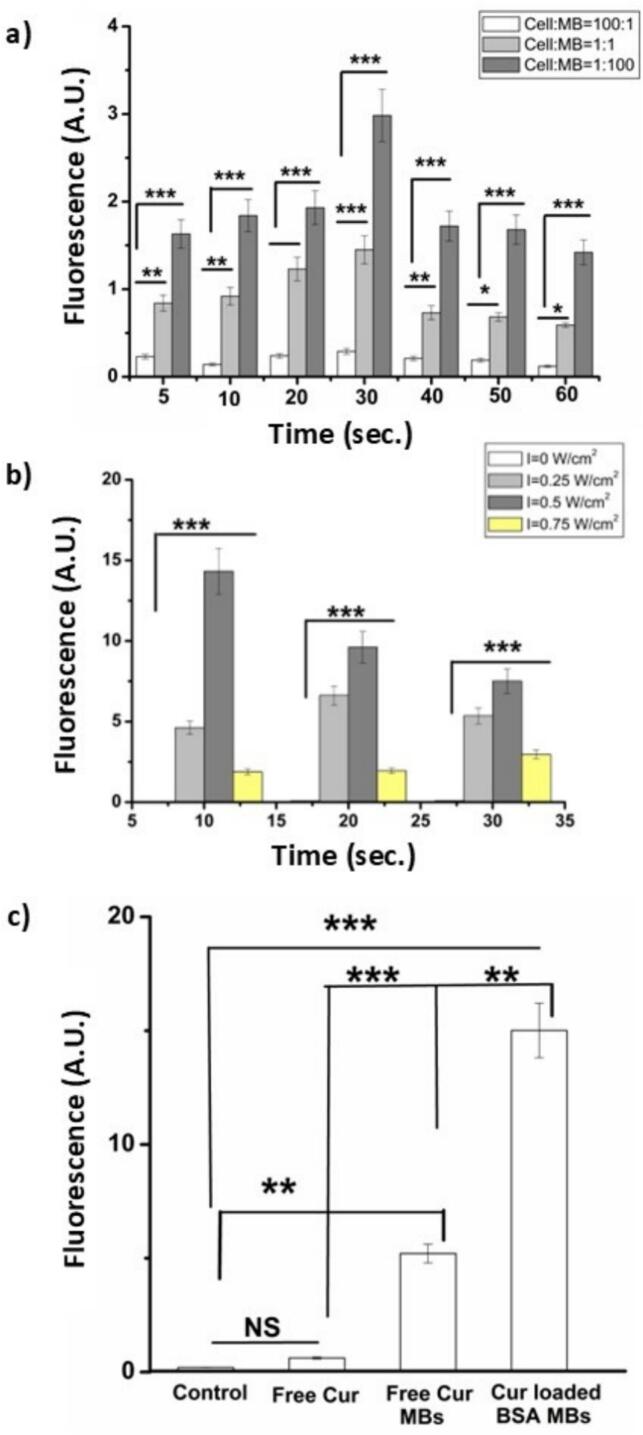
Variation in curcumin uptake, measured by the increase in fluorescence at 570 nm from curcumin, by MDAMB-231 cells when treated with CMBs (a) with varying cell:MB ratios (100:1, 1:1, and 1:100) and for different US exposure times, (b) For different US intensity and exposure time, cell:MB ratio = 1:100 and (c) compared with curcumin in DMSO with and without microbubble administration, cell:MB ratio = 1:100, US intensity = 0.5 W/cm^2^, for 10 s. The control refers to untreated cells. ***p < 0.001; **p < 0.01; *p < 0.05.

Curcumin fluoresces at 570 nm and thus any increase in fluorescence intensity at this wavelength from the cells after exposure to curcumin and washing, indicates curcumin uptake by the cells [44]. Curcumin uptake by the MDAMB-231 cells increased at all sonication exposure times as the cell-to-microbubble ratio decreased, Fig. 8(a). This is likely due to a higher curcumin payload. Lower cell:MB ratios imply more microbubbles per cell. The fluorescence values in Fig. 8(a) are relatively low, which may be due to cell death caused by the ultrasound intensity (0.75 W/cm^2^) used in this optimisation step [44]. Nevertheless, the highest uptake for curcumin was observed at the cell:MB ratio of 1:100. Therefore, the subsequent studies used this ratio. At the 1:100 cell:MBs ratio, an US intensity of 0.5 W/cm^2^, over 10 s, led to the highest drug uptake, Fig. 8(b). The cell samples had been washed, following the protocol laid out, prior to analysis. This washed out any dead cells as they could not adhere to walls of the cell culture well plates.

Based on the above findings, for the final study to optimise curcumin uptake, the following optimized parameters used were: cell:MB ratio = 1:100; US intensity = 0.5 W/cm^2^; US exposure time = 10s. Untreated MDAMB-231 cells were used as a control. Sonication of the MDAMB-231cells, with CMBs present, led to an increase in curcumin uptake (***p < 0.001), Fig. 8(c) and Table 1. Curcumin uptake by the cells was more effective with CMBs than using blank MBs co-administered with Cur dissolved in DMSO, or curcumin dissolved in DMSO alone. In the absence of microbubbles altogether, curcumin uptake was negligible for the cells. The mechanical force generated by ultrasound through interactions between the microbubbles and the cells can trigger a process known as “sonoporation.” This phenomenon is considered one of the most promising approaches for achieving spatiotemporally controlled drug delivery to targeted regions [58].

### 3.8 *In vitro* cytotoxicity: 2D and 3D cell culture models

**Fig. 9:**
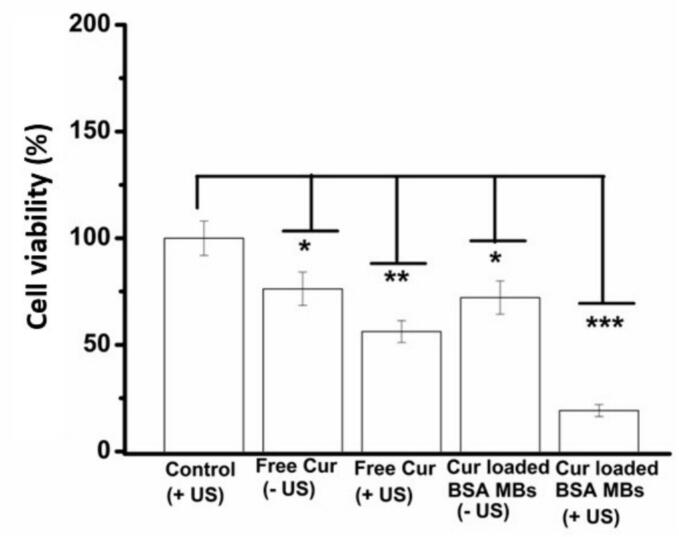
Cell viability for the MDAMB-231 cells, subjected to free curcumin dissolved in DMSO with and without ultrasound or CMBs with and without US, at a cell:MB ratio of 1:100 and a 48-hour incubation. (+US: with ultrasound; −US: without ultrasound), ***p < 0.001; **p < 0.01; *p < 0.05.

Cytotoxicity of the MBs drug delivery platform was assessed using MDA-MB-231 cells using a 2D culture model. Cellular proliferation differed significantly (*p < 0.05) when cells were treated with only free curcumin and CMBs without US compared to untreated cells, Fig. 9 and Fig. S6. However, cellular proliferation was more significantly arrested when cells were treated with free curcumin (**p < 0.01) as well as CMBs (***p < 0.001) with US. It should be noted that the optimised ultrasonication conditions with and without BSA in solution were not found to negatively impact cell viability of MDAMB-231 cells (Fig. S6). Live /dead assay supported the findings from the cellular viability assay. The highest number of dead cells were detected when cells were exposed to CMBs with US, Fig. S7.

**Fig. 10:**
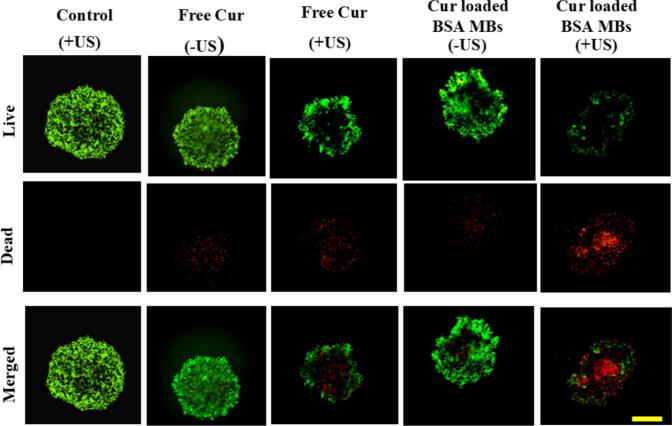
Fluorescence micrographs showing LIVE/DEAD staining of the 3D cultured MDA-MB-231 cells in the spheroid tumour models treated with free curcumin and curcumin-loaded microbubbles with and without ultrasound. Green: Live cells, Red: Dead cells. Scale bar = 1 mm. (+US: with ultrasound; −US: without ultrasound). (For interpretation of the references to colour in this figure legend, the reader is referred to the web version of this article.)

To more closely mimic *in vivo* conditions, 3D tumour spheroid models were used [59]. Cell proliferation was significantly reduced (increased number of dead cells when a tumour spheroid was treated with CMBs exposed to US when compared with the untreated 3D model), Fig. 10. An increased number of dead cells were visualized when the tumour spheroid was treated with curcumin dissolved in DMSO and exposed to US than in the untreated model, but the killing was not as efficient as with the curcumin loaded microbubbles, Fig. 10. Thus, CMBs, combined with US, significantly reduced the cell viability in a 3D tumour model. This is attributed to the more effective cellular uptake of curcumin when using CMBs. The CMBs, when exposed to US, ruptured and provided a steady exposure to curcumin.

The application of US, with MBs present, can trigger sonoporation of cell membranes [60]. This would create transient pores in the membrane which reseal within ∼20s [61]. Consequently, uptake of a drug (curcumin in our case) must occur within this brief timeframe. Sonication of MDAMB-231 cells in the presence of a curcumin solution (without the MBs) did not increase the curcumin uptake. This is because curcumin molecules must traverse both the surrounding medium and the cell cytoplasm to access the open pores very quickly. The slow diffusion process and the rapid resealing of sonoporated cell membranes contribute to an inefficient uptake of curcumin. In the study, the cell-to-CMB ratio was maintained at 1:100. At this concentration, it is highly likely that there will be multiple CMBs adjacent to any given cell. Hence, we hypothesise that in conjunction with “sonoporation”, once the CMBs burst, there will an elevated concentration of curcumin adjacent to the cells. It is hypothesised from this work that following sonoporation of the cell membrane, there is rapid diffusion of the abundant amounts of curcumin that has been released as well as minute MB shell fragments loaded with curcumin. This leads to a swift and increased uptake of curcumin by the MDAMB-231 cells. Hence, ultrasound triggered sonoporation in cell membranes facilitated uptake of the drug being delivered by the MBs. This may be a further reason for the high cell deaths in both the 2D and 3D (tumour spheroid) cultures.

## 4 Conclusion

This study employs a vortex-based hydrodynamic cavitation device to produce stable microbubbles using proteins. When preparing MBs with BSA solutions, the higher protein concentration (15 wt%) created a more stable microbubble suspension. Microbubble suspensions kept at 4 °C exhibited greater stability, compared to samples stored at room temperature, attributed to the increased flexibility of MB shells at warmer temperatures. Subsequently, a 15 wt% BSA solution was utilized to create CMBs. *In vitro* kinetics of curcumin release from the CMB surface confirmed the potential of ultrasound to trigger the release of curcumin. Curcumin uptake studies verified that curcumin loaded microbubbles, used alongside ultrasound, can enhance curcumin uptake of the targeted cells, compared to curcumin alone or co-administered with blank microbubbles. *In vitro* cell viability studies demonstrated the high effectiveness of curcumin loaded BSA MBs at reducing cellular proliferation in both 2D culture and 3D tumour spheroid models with ultrasound. The promising attributes of this innovative cavitation device, along with extended shelf life, gentle preparation conditions, and comprehensive *in vitro* evaluations with tumour spheroid models, lay the foundation for further advancements in the development of “smart microbubbles”.

## CRediT authorship contribution statement

**Promita Bhattacharjee:** Writing – review & editing, Writing – original draft, Methodology, Investigation, Formal analysis, Conceptualization. **Abhijeet H. Thaker:** Writing – original draft, Methodology, Investigation. **Pratik Kumar Patel:** Methodology, Investigation. **Vivek V. Ranade:** Writing – review & editing, Resources, Conceptualization. **Sarah P. Hudson:** Writing – review & editing, Writing – original draft, Supervision, Resources, Project administration, Funding acquisition, Conceptualization.

## Declaration of competing interest

The authors declare that they have no known competing financial interests or personal relationships that could have appeared to influence the work reported in this paper.
